# Methicillin-resistant *Staphylococcus aureus* in Europe, 1999–2002

**DOI:** 10.3201/eid1009.040069

**Published:** 2004-09

**Authors:** Edine W. Tiemersma, Stef L.A.M. Bronzwaer, Outi Lyytikäinen, John E. Degener, Paul Schrijnemakers, Nienke Bruinsma, Jos Monen, Wolfgang Witte, Hajo Grundmann, European Antimicrobial Resistance Surveillance System Participants

**Affiliations:** * National Institute for Public Health and the Environment, Bilthoven, the Netherlands;; † National Public Health Institute, Helsinki, Finland;; ‡ University Hospital Groningen, Groningen, the Netherlands;; § Robert Koch Institute, Werningerode, Germany

**Keywords:** Staphylococcus aureus, Europe, drug resistance, microbial, methicillin resistance, epidemiology, research

## Abstract

We explored the variation in proportions of methicillin-resistant *Staphylococcus aureus* (MRSA) between and within countries participating in the European Antimicrobial Resistance Surveillance System and temporal trends in its occurrence. This system collects routine antimicrobial susceptibility tests for *S. aureus*. We examined data collected from January 1999 through December 2002 (50,759 isolates from 495 hospitals in 26 countries). MRSA prevalence varied almost 100-fold, from <1% in northern Europe to >40% in southern and western Europe. MRSA proportions significantly increased in Belgium, Germany, Ireland, the Netherlands, and the United Kingdom, and decreased in Slovenia. Within countries, MRSA proportions varied between hospitals with highest variance in countries with a prevalence of 5% to 20%. The observed trends should stimulate initiatives to control MRSA at national, regional, and hospital levels. The large differences between hospitals indicate that efforts may be most effective at regional and hospital levels.

## Abstract 2

European Antimicrobial Resistance Surveillance System shows large variations in methicillin-resistant *S. aureus*.

*Staphylococcus aureus* is an important cause of community- and hospital-acquired infections. Infections caused by methicillin- or oxacillin-resistant *S. aureus* (MRSA) are mainly nosocomial and are increasingly reported from many countries worldwide (*1*). As MRSA strains are frequently resistant to many different classes of antimicrobial drugs, second- and third-line antimicrobial resistance is a growing concern (*2*). Surveillance of MRSA provides relevant information on the extent of the MRSA epidemic, identifies priorities for infection control and the need for adjustments in antimicrobial drug policy, and guides intervention programs (*3*).

In Europe, several surveillance systems collect data on MRSA (*4**,**5*). Most collect data from specific types of hospitals, for certain periods, or information related to specific antimicrobial susceptibility patterns. The only ongoing initiative that continuously monitors antimicrobial resistance in most European countries is the European Antimicrobial Surveillance System (EARSS), funded by Directorate General for Health and Consumer Protection of the European Commission. This network connects national surveillance systems and provides comparable and validated results of routine antimicrobial susceptibility tests (AST) following standardized protocols from a representative set of laboratories per country (*6*). Timely and detailed feedback is given through a freely accessible and interactive Web site (http://www.earss.rivm.nl). EARSS was established in 1998 and currently connects >600 laboratories in 28 countries, which serve >100 million people. Preliminary EARSS results showed considerable differences in the proportions of MRSA across Europe (*7**,**8*).

We report results of antimicrobial susceptibility testing of *S. aureus* blood isolates from 1999 to 2002 in Europe; these results show variation in the prevalence of MRSA, including variation in its proportions at the hospital level. To assess recent changes in the epidemiology of MRSA within countries, we also present country-specific temporal trends in the occurrence of MRSA.

## Materials and Methods

### Data Collection

Data (identification number of isolate, EARSS laboratory code, date and type of specimen, sex and age of patient, EARSS hospital code, hospital ward to which patient is admitted, result of *mecA* gene polymerase chain reaction [PCR], and susceptibility to several antimicrobial drugs, including oxacillin and vancomycin) are collected through national surveillance systems. AST results of every first *S. aureus* blood isolate per patient per quarter are submitted to the EARSS database by national data managers. After authorization by the national representatives by using standard feedback reports, national data are included in the EARSS database and become available on the Web site.

### Susceptibility Testing

Antimicrobial susceptibility is tested according to a standardized protocol (*5*). Briefly, laboratories report oxacillin susceptibility, preferably determined by an oxacillin-screening plate or an oxacillin disk-diffusion test. To confirm methicillin resistance, the minimum inhibitory concentration (MIC) for oxacillin or the presence of *mecA* gene by PCR is determined. Reporting vancomycin MIC is recommended for MRSA isolates.

Interpretative AST results (i.e., sensitive [S], intermediate [I], and resistant [R], in accordance with defined guidelines) are accepted. Most (71%) of the laboratories have adopted the guidelines of the National Committee for Clinical Laboratory Standards (NCCLS; www.nccls.org). Most guidelines agree that *S. aureus* isolates should be considered nonsusceptible (R) to oxacillin if the MIC is >4 mg/L. Lower MIC breakpoints (R if MIC >2 mg/L) are only suggested by the Deutsche Industrie-Norm (DIN) (www.din.de) and guidelines of the Swedish Reference Group for Antibiotics (SRGA) (www.srga.org).

### Data Analysis

We rejected observations lacking mandatory information (i.e., laboratory code, date of specimen, either patient identification number or month and year of birth, pathogen code, antibiotic code, or oxacillin test result [S or R]); duplicate records and repeat isolates from the same patient were also rejected. Isolates with an interpretative AST result of "R" (resistant) to oxacillin or one of its equivalents (cloxacillin, oxacillin, dicloxacillin, and flucloxacillin) were defined as MRSA. Isolates with intermediate susceptibility were not counted as MRSA and were excluded from the analyses. MRSA proportions were calculated as the number of MRSA isolates divided by the total number of *S. aureus* isolates obtained from blood cultures.

For the current analysis, data collected from January 1999 through December 2002 were used. We included only information from hospitals with data for > 20 isolates from countries reporting >100 isolates. To calculate time trends for analyses of variation between hospitals, we included only those hospitals that had participated in at least 3 consecutive years.

Univariate analyses were performed by using chi-square or *t* tests if appropriate. Country-specific trends in the occurrence of MRSA over time were analyzed by using a multivariate Poisson regression model adjusting for autocorrelation in hospitals (e.g., attributable to possible similarity in blood culturing and AST practice). We also compared countries with respect to variation between hospitals, expressed as the variance in hospital-specific MRSA proportions. To eliminate the natural dependency between variance and mean, the MRSA ratio was first transformed by power (Box-Cox) transformation according to the following formula: T(k/n) = (k/n)^λ^, where T is the transformed MRSA ratio, k/n is the resistance rate (i.e., the number of resistant isolates divided by the total number of isolates), and λ was chosen in such a way that variance was independent of the mean, i.e., λ = 0.397. The variance was further adjusted by size (in terms of number of isolates reported) of individual hospitals. Country-specific variances were then graphically displayed and compared.

## Results

From January 1999 through December 2002, EARSS received AST results of 53,264 *S. aureus* blood isolates from 27 countries (Norway does not report *S. aureus* data), including 628 laboratories serving 896 hospitals. Twenty-six countries reported AST results of >100 isolates. The current study included 50,759 isolates from 428 laboratories serving approximately 500 hospitals. Overall, 20% of these isolates were reported as methicillin resistant. A total of 295 hospitals (35,921 isolates, 19 countries) provided data for at least 3 consecutive years and were included in the time trend analyses. Table 1 describes the main characteristics of the data and the proportion of MRSA by country.

**Table 1:** Characteristics of EARSS database by countries^a,b^

Country (EARSS country code)	No. of hospitals^c^	Total no. of isolates	No. of MRSA isolates (%)	Period of participation
Austria (AT)	11	656	58 (8.8)	Jan 2000–Dec 2002
Belgium (BE)	36	2,953	696 (23.6)	Jul 1999–Dec 2002
Bulgaria (BG)	4	183	62 (33.9)	Jan 2000–Dec 2002
Croatia (HR)	6	341	125 (36.7)	Jul 2001–Dec 2002
Czech Republic (CZ)	35	2,426	142 (5.9)	Apr 2000–Dec 2002
Denmark (DK)	22	2,406	14 (0.6)	Jan 1999–Sept 2002
Estonia (EE)	3	112	1 (0.9)	Jan 2001–Dec 2002
Finland (FI)	17	1,990	19 (1.0)	Jan 1999–Dec 2002
France (FR)	24	3,376	1,117 (33.1)	Jan 2001–Dec 2002
Germany (DE)	25	3,757	600 (13.8)	Jan 1999–Dec 2002
Greece (GR)	19	1,126	500 (44.4)	Jan 1999–Dec 2001; Jul 2002–Dec 2002
Hungary (HU)	12	435	31 (7.1)	Jan 2001–Dec 2002
Iceland (IS)	1	184	1 (0.5)	Jan 1999–Dec 2002
Ireland (IE)	19	2,897	1,192 (41.2)	Jan 1999–Dec 2002
Israel (IL)	5	849	326 (38.4)	Jan 2001–Dec 2002
Italy (IT)	57	3,593	1,470 (40.9)	Jan 1999–Jun 2000; Apr 2001–Dec 2002
Luxemburg (LU)	4	214	41 (19.2)	Jan 1999–Dec 2002
Malta (MT)	1	240	105 (43.8)	Jan 2000–Dec 2002
Netherlands (NL)	45	5,359	30 (0.6)	Jan 1999–Dec 2002
Poland (PL)	8	238	42 (17.7)	Jan 2001–Dec 2002
Portugal (PT)	15	1,540	535 (34.7)	Jan 1999–Dec 2002
Slovakia (SK)	7	228	24 (10.5)	Jul 2001–Dec 2002
Slovenia (SI)	8	657	121 (18.4)	Jul 2000–Dec 2002
Spain (ES)	35	2,985	739 (24.8)	Jan 2000–Dec 2002
Sweden (SE)	54	6,071	48 (0.8)	Jan 1999–Dec 2002
United Kingdom (UK)	27	5,343	2,217 (41.5)	Jan 1999–Sept 2002
Total	500	50,759	10,256 (20.2)	

^a^EARSS, European Antibiotic Resistance Surveillance System; MRSA, methicillin-resistant *Staphylococcus aureus*. ^b^Only hospitals providing data of >20 isolates are included. ^c^According to EARSS hospital codes provided by the countries.

MRSA was more frequently isolated from men (21%) than from women (18%, p < 0.001). Patients with a blood culture positive for MRSA were older than patients with methicillin-susceptible *S. aureus* (MSSA) (mean age, 65.3 [SD 18.7] versus 58.6 [23.4], p < 0.001). The proportion of MRSA was highest among patients admitted to intensive care units (35%).

**Figure 1:**
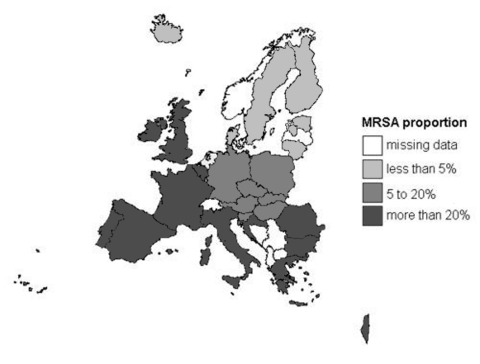
Geographic variation in proportions of methicillin-resistant *Staphylococcus aureus* (MRSA) (1999–2002).

Geographic variation is displayed in Figure 1, which shows a north-south gradient, with the lowest MRSA prevalence in northern Europe and highest prevalence in southern Europe, Israel, the United Kingdom, and Ireland. MRSA proportions varied almost 100-fold, with the lowest proportion in Iceland (0.5%) and the highest proportion in Greece (44%, Table 1).

**Figure 2:**
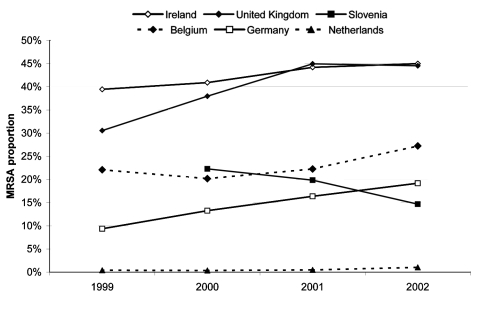
Statistically significant trends (p < 0.05) in methicillin-resistant *Staphylococcus aureus* (MRSA) proportions per year by country, 1999–2002, including hospitals participating for at least 3 consecutive years and reporting data of >20 isolates only.

Statistical analyses of country-specific time trends by Poisson regression (Table 2) showed that increases in MRSA proportions were significant in Belgium (from 22% in 1999 to 27% in 2002), Ireland (39%–45%), Germany (9%–19%), the Netherlands (0.4%–1%) and the United Kingdom (31%–45%). The proportion of MRSA decreased significantly in Slovenia only, from 22% in 2000 to 15% in 2002. The model had difficulties in estimating changes in MRSA proportion in countries with low counts of MRSA isolates, which is reflected in the very wide confidence intervals for Iceland and Bulgaria (Table 2). Relatively large year-to-year fluctuations occurred in some countries (Bulgaria, Greece, Luxembourg, Malta, and Portugal); some of these countries (Bulgaria, Luxembourg, and Malta) had low isolate counts (Table 1). Figure 2 presents significant time trends by showing MRSA proportions per country per year for 1999 through 2002.

**Table 2:** Relative change in MRSA proportion per country per year and 95% confidence intervals as calculated from Poisson regression models^a,b,c^

Country	Reported % MRSA at start	Reported % MRSA in 2002	Relative change per year, ratio	95% CI of estimated change	p value
Austria	7.0^d^	7.6	0.80	0.48 – 1.34	0.39
Belgium	22.1	27.2	1.25	1.12 – 1.41	< 0.01
Bulgaria	35.1^d^	37.7	1.11	0.59 – 2.09	0.76
Czech Republic	4.5^d^	6.2	1.15	0.89 – 1.50	0.29
Denmark	0.3	1.0	1.64	0.97 – 2.75	0.06
Finland	1.5	0.8	0.69	0.43 – 1.11	0.13
Germany	9.4	19.2	1.72	1.54 – 1.93	< 0.01
Greece	37.0	48.6	1.23	0.89 – 1.71	0.21
Iceland	0.0	0.0	0.52	0.07 – 3.67	0.51
Ireland	39.4	45.0	1.36	1.17 – 1.58	< 0.01
Italy	35.2	40.0	1.11	0.94 – 1.30	0.23
Luxembourg	15.0	18.3	1.09	0.71 – 1.67	0.70
Malta	34.7^d^	42.5	1.58	0.92 – 2.74	0.10
Netherlands	0.4	1.0	1.62	1.01 – 2.58	0.04
Portugal	39.7	38.9	0.91	0.75 – 1.09	0.32
Slovenia	22.3^d^	14.7	0.69	0.51 – 0.93	0.02
Spain	28.4^d^	23.5	1.03	0.87 – 1.21	0.74
Sweden	1.1	0.7	0.95	0.73 – 1.23	0.68
United Kingdom	30.5	44.5	1.48	1.31 – 1.66	< 0.01

^a^CI, confidence interval; MRSA, methicillin-resistant *Staphylococcus aureus*. ^b^Adjusted for autocorrelation within hospitals and for variation in the number of isolates per quarter, including only the hospitals participating for at least 3 consecutive years and reporting data of > 20 isolates. ^c^The change estimated by the model does not necessarily correspond to the overall change that can be calculated from the second and third column, this is because some trends first show an increase, followed by a decrease, or vice versa. ^d^Data from year 2000 onwards.

**Figure 3:**
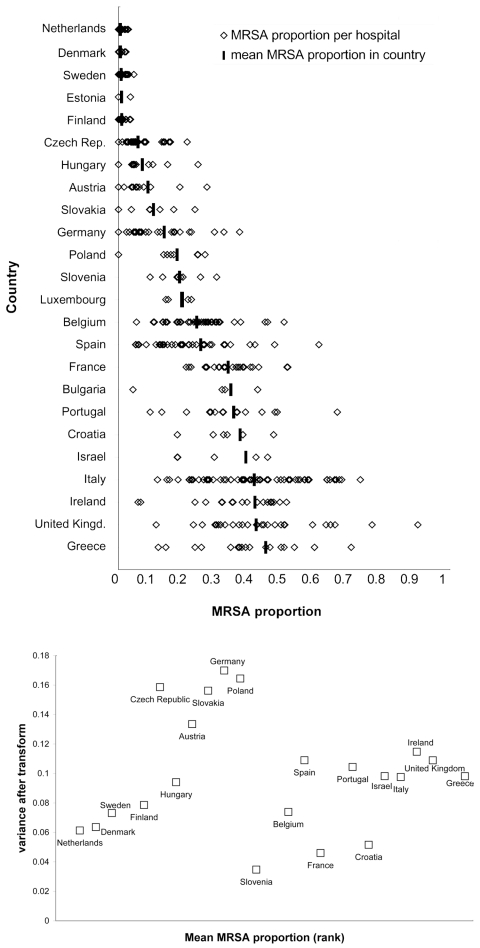
A) Variation in methicillin-resistant *Staphylococcus aureus (*MRSA) proportions between hospitals with AST results of >20 blood isolates, displayed by ranking of MRSA proportion (from lowest to highest). Only countries with more than one hospital are displayed. Hospital-specific proportions (open diamonds) are grouped per country. The solid horizontal bars represent the mean MRSA proportion per country. B) Local variation, showing the power-transformed variance being independent of the mean MRSA proportion per country, displayed by ranking of MRSA proportion (from lowest to highest). Only hospitals reporting >20 isolates are included. Countries with less than five reporting hospitals are not shown (Iceland and Malta [*1*], Estonia [*3*], Bulgaria and Luxembourg [*4*]).

Figure 3A shows regional variation in MRSA proportions within countries. Particularly high variation was identified among hospitals in Belgium, the Czech Republic, Spain, Greece, Italy, Portugal, and the United Kingdom. After applying the power transformation, the remaining variation was highest in Germany (Figure 3B), with a variance after transform of 17%. Other countries with relatively high variation in MRSA proportions (variance after transform >15%) between hospitals were Poland, the Czech Republic, and Slovakia. The highest relative variation was found in countries with MRSA proportions from 5% to 20%, with the exception of Hungary and Slovenia. A relatively high variation between hospitals was also found in countries with MRSA proportions >25%. The lowest variation between hospitals was observed for Slovenia (variance after transform, 3%), and variation was also low in France (variance after transform, 5%).

Vancomycin resistance did not occur. Intermediate susceptibility of *S. aureus* (VISA) was only reported for five isolates from France in 2001.

## Discussion

This is the first EARSS report on the prevalence of MRSA among blood isolates in 27 countries in the European region. We found that proportions of MRSA vary largely across Europe, with the highest proportions in southern and parts of western Europe and lowest proportions in northern Europe. MRSA proportions seem to be increasing in many countries. Significant increases were found for Belgium, Germany, the Netherlands, Ireland, and the United Kingdom, whereas the proportion of MRSA decreased in Slovenia. In all countries, variation between hospitals was observed. The variation between hospitals was highest in Germany and in most other countries with an MRSA prevalence of 5% to 20%. The lowest variation between hospitals was found in Slovenia.

Our results show the European situation with respect to the occurrence of MRSA in blood isolates and confirm other observations (*9**–**11*) on invasive isolates; they are also in accordance with findings of other studies with respect to demographic variables, such as sex, age, and patient ward (*9**,**12*). Although blood isolates represent the minority of clinically relevant samples, they are indicative of infection. Studies that report MRSA proportions from all sources usually include screening samples that are subject to bias because of differential screening practices. Considering hospital-acquired MRSA only seems to provide insight into the European MRSA epidemic, as the prevalence of community-acquired MRSA in Europe remains very low (0.03%–1.5%), even in countries with a high MRSA prevalence in hospitals (*13**–**17*). EARSS provides comparable data, annually validated through external quality assurance exercises, which have repeatedly confirmed a good-to-excellent concordance for identifying MRSA (*18*).

EARSS accepts susceptibility data according to clinical breakpoints (S, I, R) in agreement with international guidelines. Methicillin resistance is usually defined as having an MIC of >4 mg/L. Because of lower breakpoints (MIC >2 mg/L) defined by SRGA and DIN, this definition may have caused partial overestimation of MRSA proportions reported from Sweden (where SRGA is used in 100% of laboratories), and from Germany (where DIN is used in 59% of the participating laboratories) in comparison to other countries (*19*). However, most MRSA strains show high-level resistance to oxacillin, although low-level resistant strains are emerging (*20*). Moreover, such misclassification is unlikely to bias the country-specific temporal trends reported here. In all other countries, all laboratories agree on a single breakpoint (>4 mg/L).

We used Poisson regression modeling adjusting for autocorrelation within hospitals to test for possible time trends in MRSA proportions. This model assumes that the epidemic runs according to an *S*-curve (*21*). The results of this analysis need to be interpreted with caution, as confidence intervals are wide, especially for countries with a low number of isolates. Year-to-year fluctuations found for some countries were probably not due to changes in the case-mix, as analyses were performed on data from a constant set of hospitals in each country, but were possibly caused by random variation of low numbers of isolates (Bulgaria, Luxembourg, Malta). Since the model estimates time trends over the 4-year observation period, it did not account for such fluctuations, which should be possible by autoregressive moving average (ARIMA) modeling (*22*). However, ARIMA modeling requires at least 60 data points, which cannot be provided at this stage.

The temporal increase we found for Germany is supported by a national surveillance study carried out at regular intervals, which reported an increase of MRSA from 2% in 1992 to 21% in 2001 (*23**,**24*). Our results for the United Kingdom show that the increase in MRSA proportions, reported from 1992 through 1998 (*25**,**26*), continued until 2001, and now appears to have leveled off. This development in the MRSA epidemic reflects the curve of the number of hospitals affected by MRSA outbreaks over time, as predicted by Austin and Anderson (*21*). The same epidemic curve might apply to Ireland, although the stabilizing MRSA prevalence may also be the result of a nationwide infection control campaign (*27*). This Strategy for the Control of Antimicrobial Resistance in Ireland (SARI) follows a multidisciplinary approach, focusing on surveillance of antimicrobial resistance and use as well as infection control and stewardship of antibiotic use in the community and in hospitals. National MRSA guidelines are being updated, and the deficit in hospital staffing (laboratory surveillance scientists, infection control nurses, clinical microbiologists, and clinical pharmacists) is currently being addressed (*28*). In England, several recent initiatives have the goals of increasing awareness and encouraging efforts to control MRSA by individual hospitals. First, a mandatory surveillance program for MRSA bacteremia was launched, which included publication of MRSA diagnoses by named health trust (*29**,**30*). Second, a strategy was published to reduce healthcare-associated infection in England (*31*), which included guidelines for good hospital practice. The rise in MRSA prevalence in the Netherlands might be the result of the increase in heterogeneously resistant clones with low MICs for oxacillin (4–24 mg/L) (*32*). The effects of national infection control campaigns launched in Slovenia (J. Kolman, pers. comm.) may have had an impact. With the continuation of EARSS, we will be able to monitor any effect of such campaigns.

Variations in MRSA proportions between hospitals within the same country have been reported (*9**,**33**–**35*), but to our knowledge, this is the first attempt to quantify variation between hospitals at the national level in a European study. We showed that considerable variation in MRSA proportions exists not only between countries but also between hospitals within a country. Regional variation might be explained by different phenomena. The emergence of MRSA is largely due to dissemination of clonal strains, and temporary hospital outbreaks are typically due to clonal expansion (*36*). If stringent control measures are taken to prevent further MRSA transmission, MRSA prevalence might subsequently be reduced to sporadic levels (*12*). However, the effectiveness of MRSA control depends on several factors, such as the existence and correct application of hygiene protocols to prevent transmission (hand hygiene, isolation practices, cohorting), level of care needed by patients (indicating host susceptibility), and antimicrobial drug prescription policies (which would influence selective pressure), which might differ between hospitals in a country (*37*). As Kotilainen and colleagues showed, quick and adequate measures at the hospital level, as well as at the regional level, may be successful in containing the MRSA epidemic (*38*). Regional variation may also be explained by differences in diagnostic practice and culturing activity and random errors, which may artificially increase variation (*39*). Also, a differential case-mix attributable to differences in the level of care provided per hospital and differential referral practice may confound our estimates (*9**,**35**,**40*). However, unusually high variation in MRSA proportions between hospitals seems to occur most often in countries experiencing a current surge of MRSA. In support of this hypothesis, in general, MRSA proportions varied most in countries with increasing and intermediate (5%–20%) MRSA prevalence. These countries might have changed from equilibrium with adequate control and elimination of sporadic MRSA and might be on the verge of becoming MRSA-endemic. This stage may be characterized by abandoning strict search-and-control strategies and adopting more flexible approaches, as happened in England when MRSA prevalence was increasing in the 1990s (*41**,**42*). However, MRSA proportions were not increasing in all these countries, and variation in prevalence between hospitals was also high in countries with a high overall MRSA prevalence (>25%) (*37*). In contrast, in Slovenia, where MRSA proportions have decreased recently, variation between hospitals was low. Thus, the national campaign on infection control might have decreased not only MRSA prevalence but also the variation in MRSA proportions between hospitals.

Our database did not show vancomycin resistance; a few VISA isolates were reported from France only. This finding might be explained by the fact that EARSS collects routine data, whereas VISA will only be detected in specialized laboratories. Moreover, the clinical and epidemiologic importance of (heterogeneous) VISA remains to be clarified.

EARSS results show that MRSA proportions increased in several countries. Variation in MRSA proportions exists at international and at national levels, and regional variation seems to be highest in countries with intermediate MRSA proportions (5%–20%). Although the reasons for this phenomenon are unknown, high variation may occur in countries where the epidemiology of MRSA is in a transition period (e.g., Germany). Also in countries with a high MRSA proportion, between-hospital variation remains considerable. The large differences between hospitals indicate that initiatives may be most effective when undertaken at the local or regional level (*38*). To combat the MRSA epidemic, public health researchers and all health professionals must understand the role of hospital hygiene protocols and of antimicrobial drug policies, as well as mechanisms of regional spread of MRSA throughout hospitals. Studies that link information on MRSA guidelines, antimicrobial policies, and prescriptions with resistance rates at the level of the hospital, region, or both, may increase our understanding of the nature of the MRSA epidemic (*43*).
